# Association between weight-adjusted waist index and Hashimoto’s thyroiditis: insights from NHANES 2007–2012

**DOI:** 10.3389/fnut.2024.1520440

**Published:** 2025-01-06

**Authors:** Xiaoyong Wen, Yu Mao, Zeyu Li, Guangji Chen, Shiwei Zhou

**Affiliations:** ^1^Department of Thyroid Surgery, The Affiliated Cancer Hospital of Xiangya School of Medicine, Central South University/Hunan Cancer Hospital, Changsha, Hunan, China; ^2^Department of Thyroid Surgery, The Second Xiangya Hospital, Central South University, Changsha, Hunan, China; ^3^Department of Surgery, University Hospital, Central South University, Changsha, Hunan, China

**Keywords:** weight-adjusted waist index, central obesity, Hashimoto’s thyroiditis, thyroid peroxidase antibodies, NHANES

## Abstract

**Objective:**

While previous studies have explored the relationship between obesity and levels of thyroid autoantibodies, research using novel indicators such as weight-adjusted waist index (WWI) remains limited. This study aimed to evaluate the potential relationship between WWI and thyroid autoantibody levels, with the objective of improving our understanding of the links between central obesity and Hashimoto’s thyroiditis (HT).

**Methods:**

We conducted a cross-sectional study using data from the National Health and Nutrition Examination Survey (NHANES) cycles from 2007 to 2012. We analyzed the relationship between WWI and levels of thyroid peroxidase antibodies (TPOAb) and thyroglobulin antibodies (TgAb) through multivariate linear regression and subgroup analyses.

**Results:**

The study included 7,056 participants with an average age of 49.71 ± 17.66 years, comprising 49.18% females. Mean WWI across the cohort was 11.04 ± 0.84. Analysis revealed a significant positive association between WWI and TPOAb levels (*β*: 4.78, 95% CI: 1.52, 8.05, *p* = 0.0041), which remained consistent across all multivariate linear regression models. In contrast, no significant correlation was found between WWI and TgAb levels after adjusting for covariates. Subgroup analysis stratified by gender demonstrated a notable gender-specific effect, where the positive correlation between WWI and TPOAb levels was evident only in females (*β*: 8.13, 95% CI: 4.14, 12.12, *p* < 0.0001).

**Conclusion:**

This study used WWI as a novel indicator of central obesity and identified a strong association with HT, particularly notable in females. However, further high-quality studies are needed to confirm these findings and explore the underlying biological mechanisms.

## Introduction

Thyroid autoantibodies, including thyroid peroxidase antibodies (TPOAb) and thyroglobulin antibodies (TgAb), are crucial markers for diagnosing and managing Hashimoto’s thyroiditis (HT) (1). TPOAb targets thyroid peroxidase, an enzyme essential for thyroid hormone production, while TgAb targets thyroglobulin, the protein precursor to these hormones. Elevated levels of these antibodies indicate an autoimmune response against the thyroid gland and are commonly observed in HT (2). This autoimmune attack on thyroid tissue can ultimately lead to irreversible hypothyroidism. Furthermore, chronic inflammation and immune dysregulation create a conducive environment for carcinogenesis within thyroid tissue (3). Consequently, HT has emerged as a significant public health concern. Understanding the factors that influence TPOAb and TgAb levels can offer valuable insights into the pathogenesis of these disorders and aid in developing effective treatment strategies.

In recent years, obesity has been recognized as a systemic chronic inflammatory disease, with adipocytes playing a critical role in chronic inflammation and immune regulation (4–6). Various studies have reported a link between obesity and HT, but this relationship remains controversial (7). Traditional obesity assessment tools, such as Body Mass Index (BMI) and Waist Circumference (WC), have limitations (8). For example, BMI cannot distinguish between central and peripheral obesity and varies with age, gender, and other factors (9, 10). In 2018, a new anthropometric index, the weight-adjusted-waist index (WWI), was introduced to assess obesity (11).

The WWI offers a more precise assessment of central obesity, which is crucial for understanding associated health risks, including metabolic and cardiovascular health (11). Using this novel indicator to investigate the correlation between obesity and HT may reveal a more accurate relationship, thereby contributing to the effective management of HT. Thus, our aim is to evaluate the potential relationship between WWI and thyroid autoantibody levels using data from the National Health and Nutrition Examination Survey (NHANES) covering the years 2007 to 2012, with the objective of improving our understanding of the links between central obesity and HT.

## Materials and methods

### Study design

This cross-sectional study adhered to the guidelines outlined in the Strengthening the Reporting of Observational Studies in Epidemiology (STROBE) statement (12).

### Study population

This cross-sectional study utilized data from the National Health and Nutrition Examination Survey (NHANES) cycles from 2007 to 2012. NHANES is a national survey that evaluates the health and nutritional status of the U.S. population using a representative sample of non-hospitalized residents (13). Detailed information about the NHANES survey design can be found on the CDC website at https://www.cdc.gov/nchs/nhanes/index.htm.

**Figure 1:**
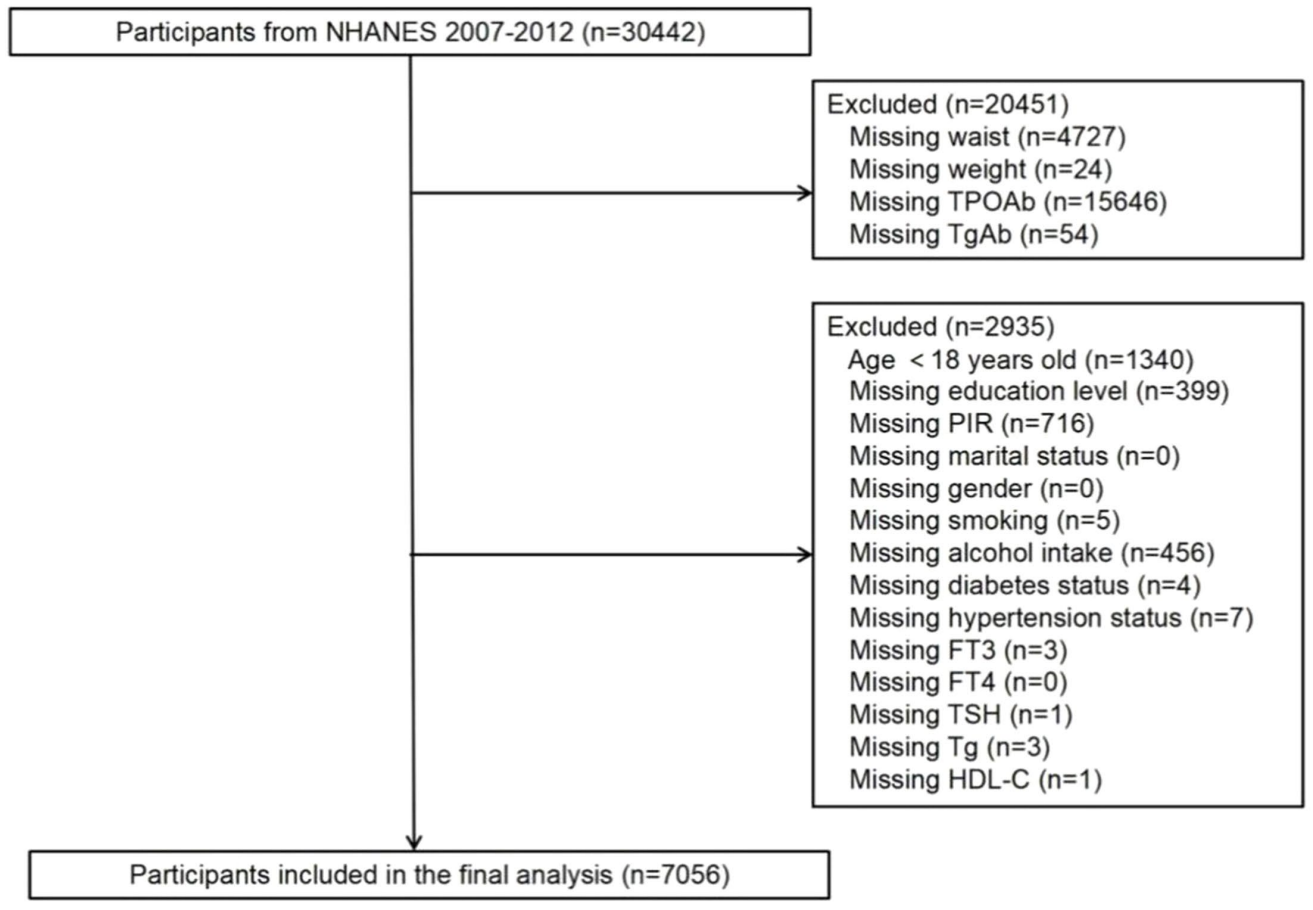
Flowchart of population included in the final analysis.

While data related to WWI (weight and waist measurements) are present in all NHANES datasets, only the dataset spanning 2007 to 2012 included comprehensive data on thyroid function. Hence, this analysis utilized data from the 2007–2012 dataset. In this cohort, 30,442 individuals were initially considered. Exclusions were applied for participants lacking data on thyroid autoantibodies (a total of 15,700 individuals, comprising 15,646 without TPOAb data and 54 without TgAb data), WWI (a total of 4,751 individuals, including 4,727 without waist circumference data and 24 without weight data), and covariates (a total of 2,935 individuals). Ultimately, the study enrolled a total of 7,056 participants, as depicted in Figure 1.

### Evaluation of central obesity

The WWI is designed to assess central obesity, calculated by dividing waist circumference (in cm) by the square root of body weight (in kg) (14). In this study, WWI was treated as the exposure variable.

### Evaluation of HT

The HT was assessed based on the levels of thyroid peroxidase antibodies (TPOAb) and thyroglobulin antibodies (TgAb) in individual serum samples. The magnitude of TPOAb and TgAb levels partly reflects the severity of autoimmune thyroid disease (15). Due to the lack of thyroid ultrasound imaging data in the NHANES dataset, this study could not evaluate HT at an imaging level.

### Selection of covariates

To enhance the accuracy of this study, we included a variety of sociodemographic and behavioral factors, along with selected biochemical markers related to thyroid function and obesity, as potential confounding variables. The included covariates were: age (years), gender (Male/Female), racial/ethnic group (Mexican American, Other Hispanic, Non-Hispanic White, Non-Hispanic Black, or Other race/multiracial), education level (Less than high school, High school, or More than high school), Poverty Income Ratio (PIR), marital status (Married or living with partner, Divorced, separated, or widowed, Never married), smoking status (indicating if the participant has smoked at least 100 cigarettes in their life: Yes/No), alcohol intake (drank at least 12 alcoholic drinks in the past year: Yes/No), diabetes status (Yes/No), hypertension status (Yes/No), levels of FT3, FT4, TSH, Tg, and High-density lipoprotein cholesterol (HDL-C). The selection of covariates was based on their established associations with both obesity and thyroid autoimmunity, ensuring that potential confounding factors were adequately accounted for. Demographic variables such as age, gender, and racial/ethnic group were included due to their influence on thyroid function and obesity prevalence. Socioeconomic factors, represented by education level and Poverty Income Ratio (PIR), were considered for their impact on health behaviors and access to healthcare. Lifestyle factors, including smoking status and alcohol intake, were selected because of their known effects on metabolic health and immune regulation. Clinical variables, such as diabetes and hypertension status, were included given their associations with chronic inflammation and thyroid dysfunction. Additionally, thyroid-related parameters (FT3, FT4, TSH, Tg) and metabolic markers like HDL-C were incorporated to provide context for the relationship between WWI and thyroid autoantibodies. This comprehensive selection ensured a robust analysis and minimized bias in evaluating the association.

### Statistical analysis

Statistical analysis adhered to survey methods and NHANES analysis guidelines. Continuous variables were presented as mean ± standard deviation (Mean ± SD), while categorical variables were expressed as percentages. The relationship between WWI and thyroid autoantibodies was evaluated using multivariate linear regression models with three levels of adjustment: unadjusted, minimally adjusted, and fully adjusted models. To further assess the association across different levels of central obesity, WWI was divided into quartiles, and separate linear regression analyses were conducted for each quartile (16). Additionally, smoothed curve fitting was employed to assess the relationship between WWI and thyroid autoantibodies, providing a graphical representation of the association. Subgroup analysis was conducted to evaluate effect modification across various subgroups, including gender, age, race, education level, smoking status, alcohol intake, diabetes status, and hypertension status. Interaction tests were applied to assess the consistency of these associations across the different subgroups. All statistical analyses were performed using R software (version 4.2) and EmpowerStats (v.2.0^1^, X&Y Solutions, Inc., Boston, MA, United States), with a two-sided *p*-value of less than 0.05 considered statistically significant.

### Ethics approval and consent to participate

All procedures conducted in studies involving human participants adhered to the ethical standards set by the institutional and/or national research committees, in line with the 1964 Helsinki Declaration and its subsequent amendments or equivalent ethical guidelines. The analyses were based on data from the National Health and Nutrition Examination Survey (NHANES). The study received approval from the Ethics Review Board of the National Center for Health Statistics. Detailed information is available on the NHANES website. Written informed consent was obtained from each participant prior to their inclusion in the NHANES database. Further details regarding the ethics application and informed consent process are also provided on the NHANES website.

## Results

### Population characteristics

Our study included 7,056 individuals with an average age of 49.71 ± 17.66 years, of whom 49.18% were female and 48.07% were Non-Hispanic White. Additional demographic data are presented in Table 1. The average WWI for all participants was 11.04 ± 0.84. The mean WWI values for each quartile (Q1–Q4) were as follows: Q1: 8.11–10.47, Q2: 10.47–11.05, Q3: 11.05–11.61, and Q4: 11.61–15.39. The mean TPOAb and TgAb values for all individuals were 20.48 ± 94.02 IU/mL and 9.94 ± 89.71 IU/mL, respectively. Both TPOAb and TgAb levels showed an upward trend across increasing WWI quartiles (TPOAb: Q1: 12.81 ± 64.43 IU/mL, Q2: 19.61 ± 86.33 IU/mL, Q3: 23.38 ± 99.57 IU/mL, Q4: 26.11 ± 117.29 IU/mL, *p* < 0.001; TgAb: Q1: 4.79 ± 48.68 IU/mL, Q2: 8.39 ± 81.31 IU/mL, Q3: 12.34 ± 103.75 IU/mL, Q4: 14.24 ± 111.40 IU/mL, *p* = 0.009). Individuals in the higher WWI quartiles tended to be older, more likely to be female, and had lower incomes and education levels. They also showed a higher prevalence of diabetes and hypertension, elevated TSH levels, and lower HDL-C levels.

**Table 1:** Basic characteristics of participants by weight-adjusted waist index quartile.

Characteristics	Weight-adjusted-waist index quartile (cm/√kg)				Overall	*P-*value
	Q1 (8.11–10.47)	Q2 (10.47–11.05)	Q3 (11.05–11.61)	Q4 (11.61–15.39)		
	*N* = 1764	*N* = 1764	*N* = 1764	*N* = 1764		
Age (years)	37.98 ± 14.75	46.98 ± 15.69	54.07 ± 16.09	59.82 ± 16.10	49.71 ± 17.66	<0.001
Gender, (%)						<0.001
Male	1,045 (59.24%)	973 (55.16%)	884 (50.11%)	684 (38.78%)	3,586 (50.82%)	
Female	719 (40.76%)	791 (44.84%)	880 (49.89%)	1,080 (61.22%)	3,470 (49.18%)	
Race/ethnicity, (%)						<0.001
Mexican American	151 (8.56%)	283 (16.04%)	333 (18.88%)	348 (19.73%)	1,115 (15.80%)	
Other Hispanic	135 (7.65%)	185 (10.49%)	210 (11.90%)	203 (11.51%)	733 (10.39%)	
Non-Hispanic White	872 (49.43%)	831 (47.11%)	814 (46.15%)	875 (49.60%)	3,392 (48.07%)	
Non-Hispanic Black	468 (26.53%)	334 (18.93%)	307 (17.40%)	262 (14.85%)	1,371 (19.43%)	
Other race/multiracial	138 (7.82%)	131 (7.43%)	100 (5.67%)	76 (4.31%)	445 (6.31%)	
Education level, (%)						<0.001
Less than high school	324 (18.37%)	416 (23.61%)	523 (29.67%)	675 (38.31%)	1938 (27.49%)	
High school	373 (21.15%)	414 (23.50%)	443 (25.13%)	417 (23.67%)	1,647 (23.36%)	
More than high school	1,067 (60.49%)	932 (52.89%)	797 (45.21%)	670 (38.02%)	3,466 (49.16%)	
PIR	2.74 ± 1.67	2.67 ± 1.66	2.57 ± 1.58	2.19 ± 1.52	2.54 ± 1.62	<0.001
Marital status, (%)						<0.001
Married or living with partner	968 (54.88%)	1,144 (64.85%)	1,174 (66.55%)	985 (55.84%)	4,271 (60.53%)	
Divorced, separated, or widowed	251 (14.23%)	354 (20.07%)	401 (22.73%)	588 (33.33%)	1,594 (22.59%)	
Never married	545 (30.90%)	266 (15.08%)	189 (10.71%)	191 (10.83%)	1,191 (16.88%)	
Smoking, (%)						<0.001
Yes	775 (43.93%)	815 (46.20%)	872 (49.43%)	882 (50.00%)	3,344 (47.39%)	
No	989 (56.07%)	949 (53.80%)	892 (50.57%)	882 (50.00%)	3,712 (52.61%)	
Alcohol intake, (%)						<0.001
Yes	1,416 (80.27%)	1,353 (76.70%)	1,228 (69.61%)	1,138 (64.51%)	5,135 (72.77%)	
No	348 (19.73%)	411 (23.30%)	536 (30.39%)	626 (35.49%)	1921 (27.23%)	
Diabetes status, (%)						<0.001
Yes	43 (2.44%)	124 (7.03%)	237 (13.44%)	428 (24.26%)	832 (11.79%)	
No	1721 (97.56%)	1,640 (92.97%)	1,527 (86.56%)	1,336 (75.74%)	6,224 (88.21%)	
Hypertension status, (%)						<0.001
Yes	269 (15.25%)	512 (29.02%)	735 (41.67%)	971 (55.05%)	2,487 (35.25%)	
No	1,495 (84.75%)	1,252 (70.98%)	1,029 (58.33%)	793 (44.95%)	4,569 (64.75%)	
TPOAb (IU/mL)	12.81 ± 64.43	19.61 ± 86.33	23.38 ± 99.57	26.11 ± 117.29	20.48 ± 94.02	<0.001
TgAb (IU/mL)	4.79 ± 48.68	8.39 ± 81.31	12.34 ± 103.75	14.24 ± 111.40	9.94 ± 89.71	0.009
FT3 (pg/mL)	3.23 ± 0.46	3.20 ± 0.73	3.15 ± 0.47	3.10 ± 0.58	3.17 ± 0.57	<0.001
FT4 (ng/dL)	0.80 ± 0.18	0.79 ± 0.15	0.80 ± 0.16	0.81 ± 0.20	0.80 ± 0.17	0.309
TSH (uIU/mL)	1.88 ± 3.60	1.86 ± 1.98	2.13 ± 3.65	2.16 ± 2.48	2.01 ± 3.02	0.002
Tg (ng/mL)	15.31 ± 27.62	14.79 ± 35.45	18.22 ± 61.10	19.61 ± 46.94	16.98 ± 44.64	0.003
HDL-C (mg/dL)	56.82 ± 17.19	51.76 ± 15.88	49.87 ± 15.18	49.42 ± 14.16	51.97 ± 15.91	<0.001

### Multiple regression analysis

We utilized three models to investigate the relationship between WWI and thyroid autoimmune antibodies (Tables 2, 3). The crude model did not adjust for any covariates. Model I adjusted for gender, age, and race. Model II adjusted for all covariates. When considering WWI as a continuous variable, there was a significant positive relationship between WWI and TPOAb, which remained statistically significant across all three models. In the crude model without controlling for any covariates, the *β* was 6.38 (95% CI 3.76, 9.00, *p* < 0.0001). Similarly, the *β* was 3.78 (95% CI 0.64, 6.93, *p* = 0.0183) in model I and the *β* was 4.78 (95% CI 1.52, 8.05, *p* = 0.0041) in model II. However, we only identified a significant positive relationship between WWI and TgAb in the crude model, where the *β* was 4.54 (95% CI 2.03, 7.04, *p* = 0.0004). After dividing WWI into quartiles, we observed that this positive relationship remained significant for WWI and TPOAb across all three models. In the crude model, higher WWI groups showed an increasing trend in TPOAb levels compared to the lowest quartile (*p* for trend <0.0001). In the fully adjusted model, for individuals in the highest quartile of WWI, each unit increase in WWI was associated with an increase in TPOAb levels by 11.08 IU/mL compared to those in the lowest quartile (*β*: 11.08, 95% CI: 3.75, 18.41). Similarly, the association between WWI quartiles and TgAb was statistically significant only in the crude model (*p* for trend = 0.0007), where for individuals in the highest quartile of WWI, each unit increase in WWI was associated with an increase in TgAb levels by 9.45 IU/mL compared to those in the lowest quartile (*β*: 9.45, 95% CI: 3.53, 15.37).

**Table 2:** The associations between weight-adjusted-waist index and TPOAb.

	Crude Model		Model I		Model II	
Exposure	Crude *β* (95% CI)	*P-*value	Adjusted *β* (95% CI)	*P-*value	Adjusted *β* (95% CI)	*P-*value
WWI (continuous)	6.38 (3.76, 9.00)	<0.0001	3.78 (0.64, 6.93)	0.0183	4.78 (1.52, 8.05)	0.0041
WWI (quartile)						
Q1	Reference		Reference		Reference	
Q2	6.81 (0.61, 13.00)	0.0314	5.03 (−1.34, 11.40)	0.1215	7.44 (1.19, 13.69)	0.0197
Q3	10.57 (4.37, 16.77)	0.0008	7.45 (0.73, 14.17)	0.0299	8.86 (2.13, 15.58)	0.0099
Q4	13.30 (7.10, 19.50)	<0.0001	7.85 (0.70, 15.00)	0.0313	11.08 (3.75, 18.41)	0.0031
*P* for trend	<0.0001		0.0266		0.0037	

The crude model did not adjust for any covariates. Model I: gender, age, and race were adjusted. Model II: gender, age, race, education level, PIR, marital status, smoking, alcohol intake, diabetes status, hypertension status, FT3, FT4, TSH, Tg, and HDL-C were adjusted.

**Table 3:** The associations between weight-adjusted-waist index and TgAb.

	Crude model		Model I		Model II	
Exposure	Crude *β* (95% CI)	*P-*value	Adjusted *β* (95% CI)	*P-*value	Adjusted *β* (95% CI)	*P-*value
WWI (continuous)	4.54 (2.03, 7.04)	0.0004	1.07 (−1.93, 4.08)	0.4848	1.02 (−2.19, 4.23)	0.5319
WWI (quartile)						
Q1	Reference		Reference		Reference	
Q2	3.60 (−2.32, 9.52)	0.2329	0.72 (−5.37, 6.81)	0.8157	0.95 (−5.21, 7.10)	0.7628
Q3	7.55 (1.63, 13.47)	0.0124	2.55 (−3.88, 8.98)	0.4367	2.30 (−4.32, 8.92)	0.4966
Q4	9.45 (3.53, 15.37)	0.0018	2.23 (−4.60, 9.07)	0.5225	2.31 (−4.91, 9.52)	0.5304
*P* for trend	0.0007		0.4562		0.4905	

The crude model did not adjust for any covariates. Model I: gender, age, and race were adjusted. Model II: gender, age, race, education level, PIR, marital status, smoking, alcohol intake, diabetes status, hypertension status, FT3, FT4, TSH, Tg, and HDL-C were adjusted.

### Linear correlation between WWI and thyroid autoimmune antibodies

**Figure 2:**
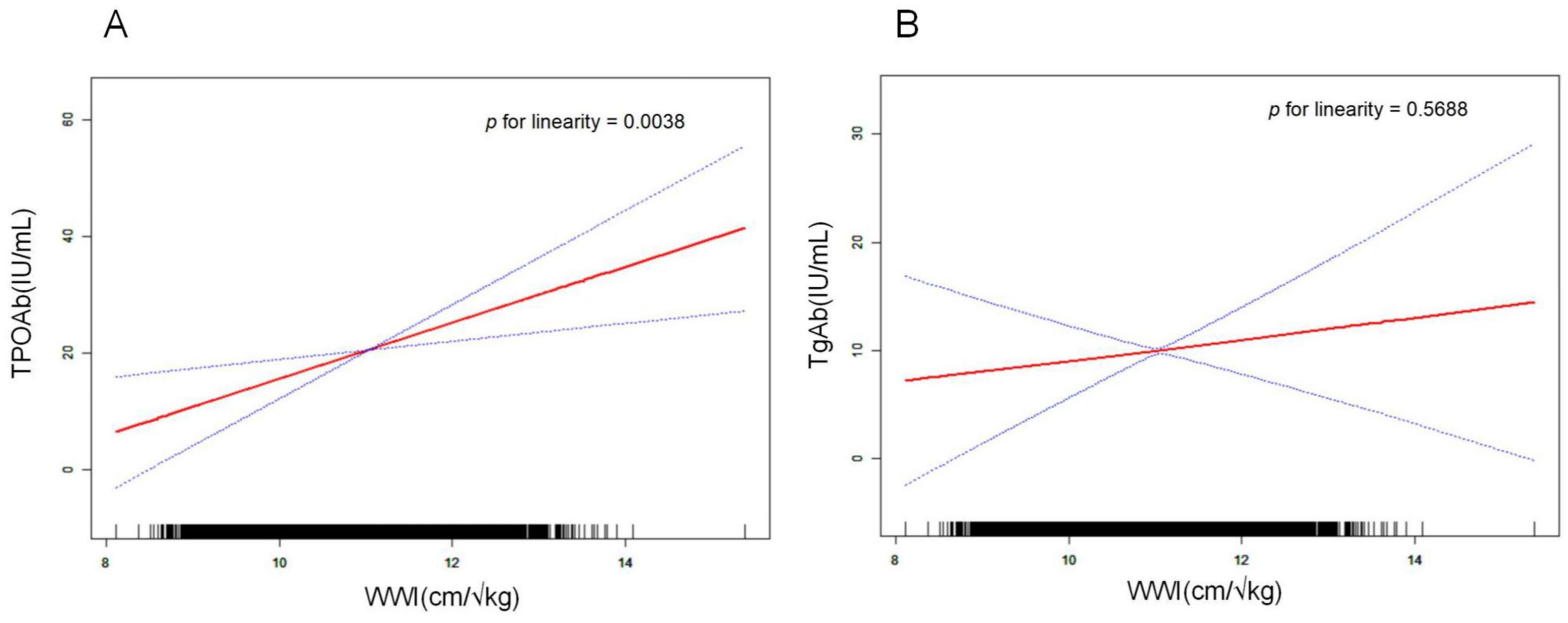
Relationship between WWI and thyroid autoantibodies. The natural spline curve shows a linear relationship between WWI and thyroid autoantibodies. The area between the blue dashed lines is considered to be the 95% confidential interval. Each read dot reveals the thyroid autoantibody levels corresponding to the WWI value, forming a continuous fitted curve. Age, gender, race, education level, PIR, marital status, smoking, alcohol intake, diabetes status, hypertension status, FT3, FT4, TSH, Tg, and HDL-C were adjusted. **(A)** Linear correlation between WWI and TPOAb (*p* for linearity = 0.0038). **(B)** Linear correlation between WWI and TgAb (*p* for linearity = 0.5688).

A smooth curve fitting was used to describe the linear relationship between WWI and thyroid autoantibodies (Figures 2A,B). Smooth curves were constructed based on model II. Results showed that WWI was linearly positively correlated with TPOAb levels (*p* = 0.0038). However, the linear relationship between WWI and TgAb (*p* = 0.5688) was less pronounced than its relationship with TPOAb.

### Subgroup analysis

**Figure 3:**
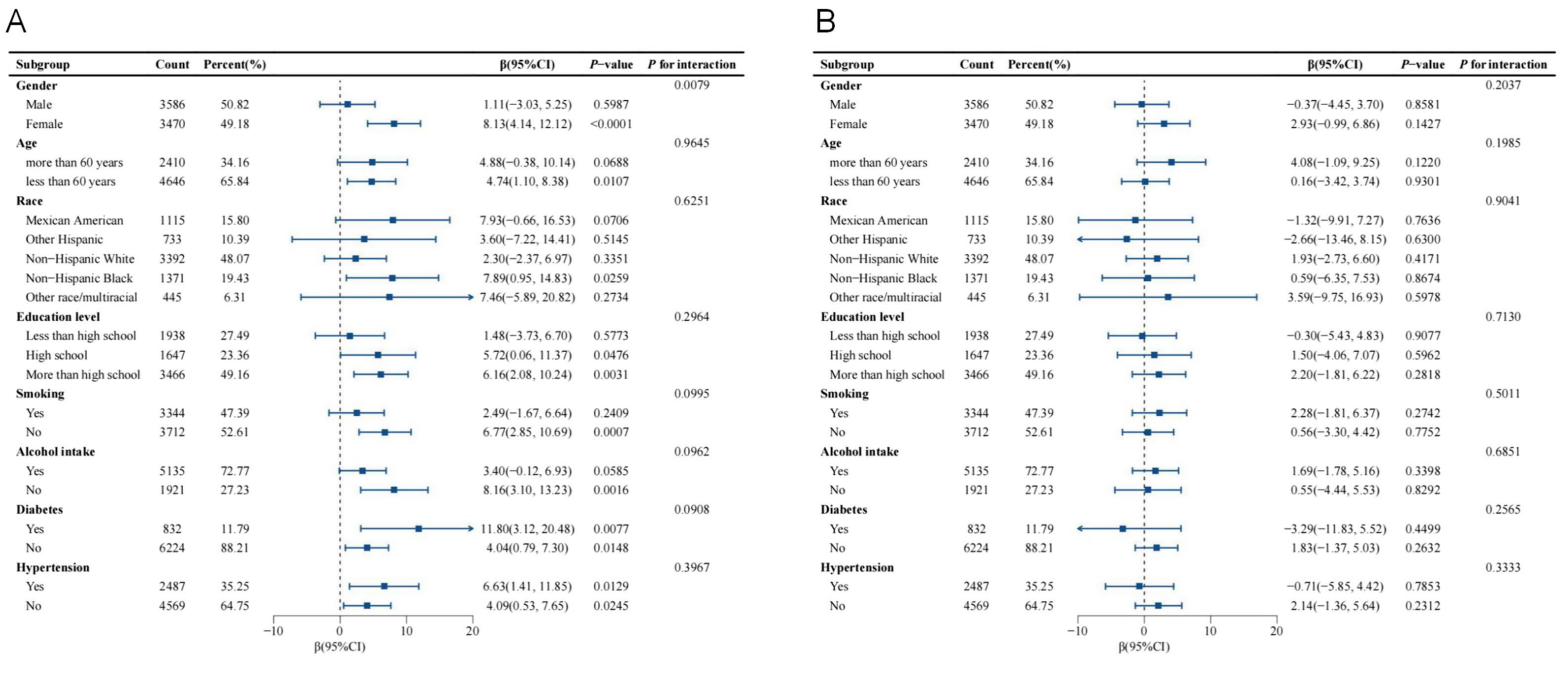
Subgroup analysis of the association between WWI and thyroid autoantibodies. Age, gender, race, education level, PIR, marital status, smoking, alcohol intake, diabetes status, hypertension status, FT3, FT4, TSH, Tg, and HDL-C were adjusted. **(A)** Subgroup analysis of the association between WWI and TPOAb. **(B)** Subgroup analysis of the association between WWI and TgAb.

As shown in Figures 3A,B, subgroup analysis and interaction tests were conducted to assess the consistency of the relationship between WWI and thyroid autoantibody levels across various subgroups. After adjusting for all covariates, significant differences in the association between WWI and TPOAb levels were found across different gender groups (*p* for interaction = 0.0079). Notably, this significant positive correlation was observed only in the female subgroup and not in the male subgroup. For female individuals, each unit increase in WWI was associated with an increase in TPOAb levels by 8.13 IU/mL (*β*: 8.13, 95% CI: 4.14, 12.12, *p* < 0.0001). Other stratified factors such as age, race, education level, smoking, alcohol intake, diabetes status, and hypertension status did not significantly influence the positive association between WWI and TPOAb levels.

## Discussion

Many scholars have endeavored to elucidate the relationship between obesity and thyroid autoantibodies levels. Meta-analyses have indicated a positive correlation between obesity and hypothyroidism, HT, and TPOAb levels, incorporating data from 22 studies that commonly used BMI as the standard measure of obesity (7). However, a study conducted in an Asian female population found no correlation between BMI and TPOAb (17), highlighting the controversy in using BMI to evaluate the relationship between obesity and thyroid autoantibodies levels. In fact, thyroid autoantibodies levels may be more closely related to metabolic disorders (18). Compared to BMI, WC, and ABSI (A Body Shape Index), WWI may serve as a superior indicator of metabolic status (14, 16, 19, 20). It has been positively associated with overall mortality, cardiovascular disease mortality, hypertension, and type 2 diabetes. Moreover, when used in conjunction with BMI, WWI can better predict the risk of developing cardiovascular metabolic diseases (11). Therefore, we conducted a cross-sectional study using the NHANES database to explore the relationship between WWI and thyroid autoantibodies levels.

The study results indicate that, after adjusting for all confounding factors, WWI was significantly positively correlated with TPOAb but not with TgAb. TPOAb is a more critical marker for HT compared to TgAb (21, 22), as 90% of patients with HT exhibit elevated serum TPOAb levels (23). Consequently, TPOAb is commonly considered the primary marker for evaluating and monitoring HT. Elevated serum TPOAb levels signify an aggressive immune attack on thyroid tissue (24). Additionally, activated lymphocytes may migrate from the thyroid to distant tissues, exacerbating the immune response and inflammation (25). In patients with HT and TPOAb levels exceeding 1,000 IU/mL, inflammation-related cytokines such as interferon-gamma and tumor necrosis factor-alpha are significantly increased, leading to systemic chronic inflammatory responses (26). This persistent immune response against thyroid tissue eventually results in irreversible hypothyroidism. Interestingly, hypothyroidism in obese patients has been shown to be a secondary response to obesity, and weight loss induced by bariatric surgery significantly reduces moderately elevated TSH levels and suppresses hypothyroidism. The degree of suppression improves over time post-surgery and correlates significantly with reductions in BMI (27). Therefore, reducing TPOAb levels by regulating WWI may effectively control thyroid inflammation, systemic inflammation, and hypothyroidism.

In further subgroup analysis, we observed that gender influenced the correlation between WWI and TPOAb levels. The significant positive correlation was evident only in the female group, with no such association found in males. Indeed, the relationship between obesity, thyroid function, and HT exhibits gender-specific differences (28). Studies have shown a positive correlation between obesity and hypothyroidism in Chinese women, whereas this correlation is not observed in men (17). Another study indicated a positive correlation between TSH and BMI specifically in obese women with positive thyroid autoantibodies (29). Our findings suggest that WWI may more significantly impact TPOAb levels in women compared to men. However, the specific mechanisms underlying these gender differences remain unclear. Potential mechanisms may include differences in fat distribution, the interplay between hormonal regulation and the immune system, and metabolic syndrome between genders (30). Women tend to accumulate fat in the lower abdomen and hip regions, while men predominantly accumulate fat in the abdominal area (31). Such differences in fat distribution may influence thyroid function and immune responses.

Estrogen, a key hormonal factor, appears to play a pivotal role in modulating immune system activity (32). Studies indicate that estrogen can exacerbate autoimmune responses in the thyroid among obese women by enhancing B and T cell activity, leading to excessive production of thyroid autoantibodies, including TPOAb (33). Furthermore, estrogen can directly influence thyroid function by binding to receptors in thyroid epithelial cells, thereby affecting their activity (34). It can also synergize with pro-inflammatory cytokines, increasing the risk of developing HT (35).

The relationship between gender differences and metabolic syndrome offers another potential explanation. Metabolic syndrome, which includes conditions such as insulin resistance and hyperlipidemia, is strongly associated with HT. Women with central obesity are generally more prone to metabolic syndrome, and insulin resistance may amplify thyroid tissue damage through pro-inflammatory pathways (36, 37). However, these mechanisms remain speculative and require further investigation to validate these hypotheses and deepen our understanding of the gender-specific effects of WWI on thyroid autoimmunity.

Thyroid dysfunction has long been associated with obesity, initially thought to be a consequence of thyroid hormone deficiency. However, treatment of hypothyroidism typically results in only modest weight loss (less than 10%), suggesting that severe obesity is generally not secondary to thyroid dysfunction (38). Instead, evidence suggests a bidirectional causal relationship between obesity and hypothyroidism, with HT potentially serving as a critical intermediary (37, 39–41). Central obesity, as reflected by a high WWI, may influence immune system activation and contribute to the development of HT (42). Adipose tissue in central obesity acts not only as an energy reservoir but also as a metabolically active endocrine organ, secreting various bioactive substances, including leptin, adiponectin, and pro-inflammatory cytokines (43, 44). Elevated leptin levels, in particular, play a central role in immune regulation by modulating T-cell activation and proliferation, which can amplify immune responses and potentially drive the progression of thyroid autoimmunity (45, 46).

Chronic low-grade inflammation, a hallmark of obesity, further compounds these effects (47). Pro-inflammatory cytokines such as TNF-*α* and IL-6 exacerbate systemic inflammation, potentially impairing thyroid epithelial cells and dysregulating immune responses (48, 49). Moreover, metabolic dysfunctions commonly associated with central obesity, including insulin resistance and oxidative stress, may create a pro-inflammatory environment that heightens the risk of immune-mediated damage to thyroid tissue (50). Together, these mechanisms underscore the intricate interplay between central obesity, chronic inflammation, and the development of HT, offering valuable insights into the pathophysiology of obesity-related thyroid autoimmunity.

However, we must acknowledge several limitations of this study. Firstly, due to its cross-sectional design, it is not possible to establish a definitive causal relationship between WWI and thyroid autoantibodies. Secondly, the study could not account for all potential covariates. Thirdly, the diagnosis of HT typically requires a combination of thyroid function tests, thyroid autoantibodies, ultrasound findings, clinical symptoms, and pathological results. Since the NHANES database lacks relevant data, our study could only examine the relationship between WWI and thyroid autoantibodies, without directly studying the association between obesity and HT. Lastly, using the NHANES dataset, which is specific to the U.S. population, may limit the generalizability of our findings globally.

Despite the above limitations, this study holds significant clinical value. To begin with, the findings support WWI as an effective tool for assessing obesity and its associated health risks, particularly in evaluating the risk of thyroid autoimmune diseases. Moreover, controlling central obesity may help mitigate thyroid autoimmune responses, offering new clinical approaches for the prevention and management of thyroid inflammation and dysfunction. Finally, the observed gender differences in the relationship between central obesity and thyroid autoimmunity provide important insights for designing personalized clinical interventions tailored to individual risk profiles.

## Conclusion

In conclusion, our study demonstrates a significant association between WWI, an indicator of central obesity, and HT, particularly in women. However, to establish causality and better understand the mechanisms involved, higher-level prospective studies are warranted. Longitudinal research would provide valuable insights into how central obesity influences HT over time, potentially guiding more effective preventive and therapeutic strategies for managing autoimmune thyroid diseases.

## Data Availability

The original contributions presented in the study are included in the article/supplementary material, further inquiries can be directed to the corresponding authors.

## Footnotes

^1^ http://www.empowerstats.com
